# Community respiratory viral metrics to inform masking in healthcare settings: A regional consensus approach

**DOI:** 10.1017/ice.2024.10

**Published:** 2024-06

**Authors:** Eric J. Chow, Lawrence Lee, Jennifer Lenahan, Sargis Pogosjans, Christopher Baliga, Mary Fairchok, John B. Lynch, John Pauk, Francis X. Riedo, Paul Thottingal, Danielle M. Zerr, Nigel Turner, James Lewis, Vicki Sakata, Jeffrey S. Duchin

**Affiliations:** 1 Public Health–Seattle & King County, Seattle, Washington; 2 Division of Allergy and Infectious Diseases, Department of Medicine, University of Washington, Seattle, Washington; 3 Department of Epidemiology, University of Washington, Seattle, Washington; 4 Section of Infectious Diseases, Virginia Mason Medical Center, Seattle, Washington; 5 Pediatric Infectious Diseases, Mary Bridge Children’s Hospital, Multicare Health System, Tacoma, Washington; 6 Infectious Disease, Swedish-Providence Health Services, Seattle, Washington; 7 EvergreenHealth, Kirkland, Washington; 8 Communicable Diseases and Organizational Preparedness, Kaiser Permanente Washington, Seattle, Washington; 9 Division of Infectious Diseases, Seattle Children’s Hospital, Seattle, Washington; 10 Department of Pediatrics, University of Washington, Seattle, Washington; 11 Tacoma-Pierce County Health Department, Tacoma, Washington; 12 Snohomish County Health Department, Everett, Washington; 13 Northwest Healthcare Response Network, Tukwila, Washington

*To the Editor—*Coronavirus disease 2019 (COVID-19) has focused our attention on the immediate and long-term health complications of respiratory viral infections. Public health strategies now must contend with evolving severe acute respiratory coronavirus virus 2 (SARS-CoV-2) community circulation in addition to the morbidity and mortality posed by endemic respiratory viral infections. During the 2022–2023 season, seasonal influenza, and respiratory syncytial virus (RSV) epidemics combined with the ongoing burden of SARS-CoV-2 infections made clear the challenges communities and healthcare systems face moving forward as well as the need to implement comprehensive respiratory virus strategies to protect people who are most vulnerable to complications. Using face masks, and the policies supporting their use, continue to play a key role in current healthcare practices to prevent nosocomial respiratory viral infections.

The use of face masks has reduced transmission of SARS-CoV-2 and other respiratory viral pathogens.^1^ In healthcare settings, universal masking policies decreased risk of healthcare-associated respiratory viral infections as part of a multilayered approach,^2^ safeguarding the health of patients and healthcare workforce. Severe COVID-19 outcomes continue to occur among vulnerable patients and healthcare facilities face ongoing workforce shortages. Masking policies remain important in preventing infections, especially when the community respiratory viral burden increases.^3^ Acknowledging the importance of masking policies, the Northwest Healthcare Response Network, a regional healthcare coalition, in collaboration with public health agencies, convened a face-mask work group of healthcare facilities in Snohomish, King and Pierce County, the 3 most populous Washington counties. We developed a regional consensus policy for universal use of face masks in health care based on emergency department (ED) visits for COVID-19, influenza, and RSV^4^ responsible for substantial annual respiratory viral disease burden. Although different policy approaches could be considered,^3^ the use of local community burden measures to determine when universal use of face masks in health care would be required for patient care areas allows healthcare facilities to justify and communicate these policies based on local disease activity and transmission risk.

When considering community measures, data should be timely, provide guidance ahead of increased community infections and be simple to message to stakeholders as the basis for why and when universal face-mask requirements would be implemented. Data sources that were regionally available were prioritized. Our public health agencies evaluated the use of syndromic surveillance ED data as a possible measure. In Washington state, syndromic surveillance is conducted through the Rapid Health Information Network (RHINO)^5^ and the Centers for Disease Control and Prevention’s National Syndromic Surveillance Program (CDC NSSP). All nonfederal Washington State EDs are required to report healthcare visit data into this system. These data include standardized pathogen discharge diagnosis codes^6^ that can be tracked for trends driven by infections in the community. ED discharge diagnoses (ED visits) for COVID-19, influenza, and RSV showed comparable timing to community burden trends reflected by laboratory test reporting^7^ before and during the COVID-19 pandemic and thus could serve as an early indicator of respiratory viral activity.

**Figure 1.**
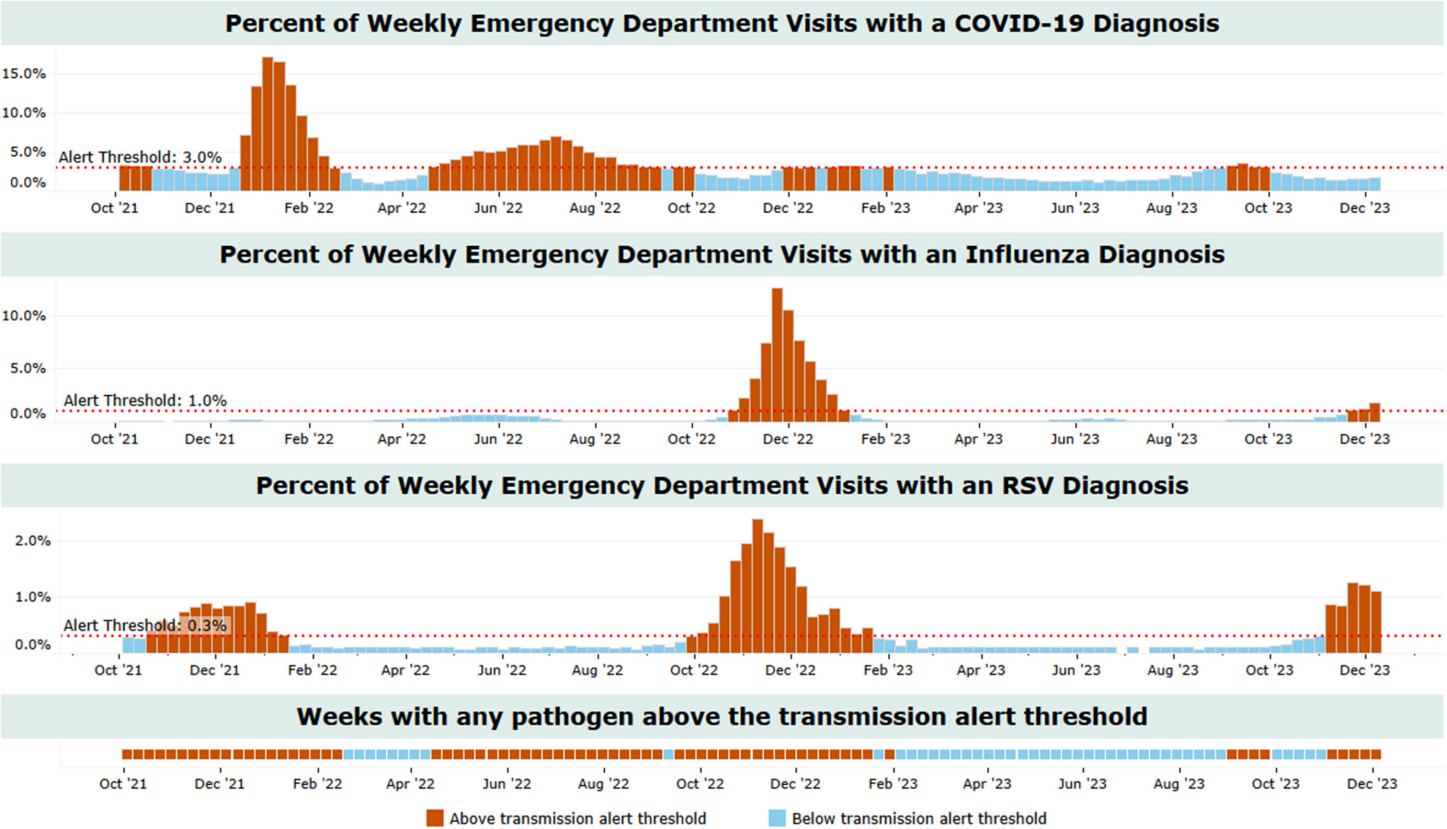
Emergency department visit trends and threshold alerts for COVID-19, influenza and respiratory syncytial virus, King County, Washington. Boxes represent 1 week.

We calculated alert thresholds for each virus using the moving epidemic method (MEM) to indicate the period when universal masking would be implemented.^8^ The MEM is a validated mathematical approach endorsed by the World Health Organization in their global epidemiological surveillance standards for influenza^9^ using epidemic trends from prior seasons. The MEM calculates a point that differentiates periods of lower community viral circulation from time points of increased activity (Fig. 1).

The work group established criteria using community respiratory viral activity published weekly by regional local public health departments to prompt activation of universal use of face masks in patient care areas. In addition to ED visit trends for COVID-19, influenza and RSV, the CDC COVID-19 hospital admission levels were also included as a criterion for activation of universal use of face masks in patient care areas.^4^ Although COVID-19 hospitalizations are likely a lagging indicator relative to ED visit trends, they were added to ensure consistency with the CDC recommendations for universal source control.^10^ Face masks are required by the time at least 1 pathogen reaches or exceeds the ED visit transmission alert threshold or if CDC COVID-19 hospital admission levels reached or exceeded 10 new COVID-19 hospital admissions per 100,000 population (7-day total) by county (“medium”), whichever occurs first. Healthcare organizations have the flexibility to use other criteria to inform the need for universal use of face masks in facilities earlier than what the established criteria would indicate, including facility-level trends in percent positivity from internal laboratory reports, COVID-19 patient census, healthcare facility outbreak activity, limitations in healthcare facility staffing capacity, or other healthcare facility metrics. The policy calls for universal use of face masks to continue until ED visit trends are below the transmission alert thresholds for all 3 pathogens and CDC COVID-19 hospital admission levels are below “medium” for at least 2 consecutive weeks.

Our approach demonstrates how face-mask policies could be implemented based on readily available local data. The ubiquity of syndromic surveillance allows the use of ED visit data for healthcare facilities seeking to adopt a similar face-mask strategy, although regional variation in data, including differences in health behavior, healthcare access, demographics, and epidemiology, may require local modifications. In our region, some healthcare facilities from counties with fewer ED facilities have referred to data from a neighboring county. We also acknowledge the novel application of MEM to SARS-CoV-2, and this methodology will require reassessment over time. This research highlights the importance of ongoing surveillance and epidemiological capacity in local public health agencies and integrated disease control strategies through partnerships with healthcare systems. Ongoing research will evaluate these metrics and refine optimal thresholds for action. Our regional face-masking consensus models how local public health and healthcare systems can work together to nimbly address a quickly evolving public health challenge.
